# Tackling Oral Giants: A Case of Verrucous Carcinoma Involving the Buccal Mucosa and Maxilla

**DOI:** 10.7759/cureus.80645

**Published:** 2025-03-16

**Authors:** Kshitij Bang, Ramakrishna Shenoi, Vrinda Kolte, Karishma D Jadhav, Hrushikesh K Malikar

**Affiliations:** 1 Oral and Maxillofacial Surgery, VSPM's Dental College and Research Centre, Nagpur, IND

**Keywords:** anterolateral thigh (alt) flap, mandibulectomy, maxillectomy, ulcero-proliferative lesion, verrucous carcinoma, wide local excision (wle)

## Abstract

Verrucous carcinoma (VC) is an uncommon, well-differentiated variant of squamous cell carcinoma (SCC) with a locally aggressive yet non-metastatic nature. It primarily affects the oral cavity, particularly the buccal mucosa, lips, and hard palate. Clinically, it presents as an exophytic, warty, or cauliflower-like lesion that can be misdiagnosed due to its resemblance to non-neoplastic ulcerative conditions. Histopathologically, VC exhibits pronounced keratinization, hyperplastic epithelium, and a test-tube-like growth pattern without significant nuclear atypia or metastasis. This case report describes a 41-year-old male presenting with a persistent ulcero-proliferative lesion diagnosed as VC. Surgical management included wide local excision, supra-omohyoid neck dissection, and partial maxillectomy with marginal mandibulectomy, utilizing a lower lip split apron flap for optimal access and minimal scarring. The defect was reconstructed with an anterolateral thigh (ALT) flap. The case highlights the necessity for precise diagnosis, an interdisciplinary approach, and the importance of surgical strategies to achieve optimal treatment outcomes in VC patients.

## Introduction

Verrucous carcinoma (VC) is an exogenous variant of well-differentiated squamous cell carcinoma (SCC) with specific morphologic, cytokinetic, and clinical features, which was first described by Ackerman in 1948 [1]. It typically affects the mucosa of the lips, oropharynx, and larynx [2,3]. Oral VC typically appears as an exophytic, broad-based lesion with a cauliflower-like warty surface. Clinically, it appears as a papillary mass with a grayish-white or red hue, distinguishing it from the typical ulcerated nodules seen in squamous cell tumors [2]. Although it is a locally aggressive tumor, it does not have a tendency to metastasize to regional lymph nodes or distant sites [4]. It grows slowly, often attaining considerable size before being noticed by the patient.

Oral VC accounts for 0.57-16.08% of oral SCC [5-7] and is predominantly seen in males, with the reported mean age at diagnosis between 49 and 69.5 years [7-9]. A variety of factors are associated with the complex etiology of oral VC, which include smoking, alcohol, and areca nut chewing. There also exists evidence of the ambiguous pathogenic role of the human papillomavirus (HPV) [10]. A stereology study by Liu et al. has described a classification first given by Tang et al. as oral VC classified into three types: exogenic, cystoid, and infiltrative, based on clinical manifestations and prognosis [11]. The exogenic type of oral VC is characterized by slow tumor development, cauliflower-like warty lesions, and exophytic growth. In contrast, the other two types of oral VC grow rapidly and form a white, dry keratosis resembling bean dregs [12].

Histologically, VC is characterized by wide and elongated rete ridges that appear to "push" into the underlying connective tissue and the presence of abundant para-keratin plugs, pronounced keratinization, well-differentiated tissue, and minimal atypia [2,10]. Due to the diverse range of causative factors and varied presentation, diagnosing and treating oral ulcerative lesions can be quite challenging. In the early stages, these cancerous ulcers can be mistaken for other pathological conditions, such as proliferative verrucous leukoplakia and verrucous hyperplasia, and are frequently misdiagnosed as non-neoplastic ulcerative lesions. Ulcers persisting for two weeks or more are often challenging to treat due to their chronic nature and resistance to various treatments [5]. The diagnosis of oral VC is based on a patient's history, clinical manifestation, and histopathologic features of the lesion [9]. Computed tomography (CT) and/or magnetic resonance imaging (MRI) are helpful in determining the local extent of the lesion with potential invasion to surrounding structures and in excluding tumors that spread to regional lymph nodes [13]. Although many different molecules associated with the diagnosis, tumor progression, and prognosis of oral VC have been proposed, a reliable and effective biomarker has still not been identified [13]. For VC, surgery is usually the first line of treatment. Radiation is rarely used as a standalone treatment or in conjunction with surgical excision [9,14].

This case report aims to provide insight into the management of VC of the oral cavity through a unique surgical approach that results in optimum outcomes with minimal scarring for the patient, as the literature lacks information on such approaches used for treating locally aggressive tumors.

## Case presentation

A 41-year-old male patient reported to the Department of Oral and Maxillofacial Surgery with the chief complaint of pain and growth in the upper right back region of the jaw for one year. The ulcero-proliferative growth was gradual in onset and slowly increased in size over the period of one year. There was a history of a burning sensation associated with the consumption of hot and spicy food and difficulty in mastication. The patient also gave a history of pain associated with the growth. No history of previous ulcerations, bleeding, or difficulty in swallowing was reported. There was no history of recurrent trauma due to any sharp teeth or restorations. No history of similar growths was noticed elsewhere in the body. The patient had visited multiple hospitals with the same complaint and underwent multiple punch biopsies. Biopsy reports had three varied conclusions: verrucous hyperplasia with an inability to rule out SCC, squamous hyperplasia, and VC. The patient had no other relevant medical or dental history contributing to this case. Additionally, the patient reported a five-year history of consuming areca nut and tobacco approximately three times daily.

On extraoral examination, the face was slightly asymmetrical due to mild swelling noted with the right cheek. The swelling was diffuse, extending supero-inferiorly from 1 cm above the ala tragus line up to 1 cm above the right corner of the mouth and anteroposteriorly from the right corner of the mouth up to 2 cm anterior to the right angle of the mandible. The submandibular and submental lymph nodes were non-palpable and non-tender. The mouth opening was reduced to 15 mm.

**Figure 1:**
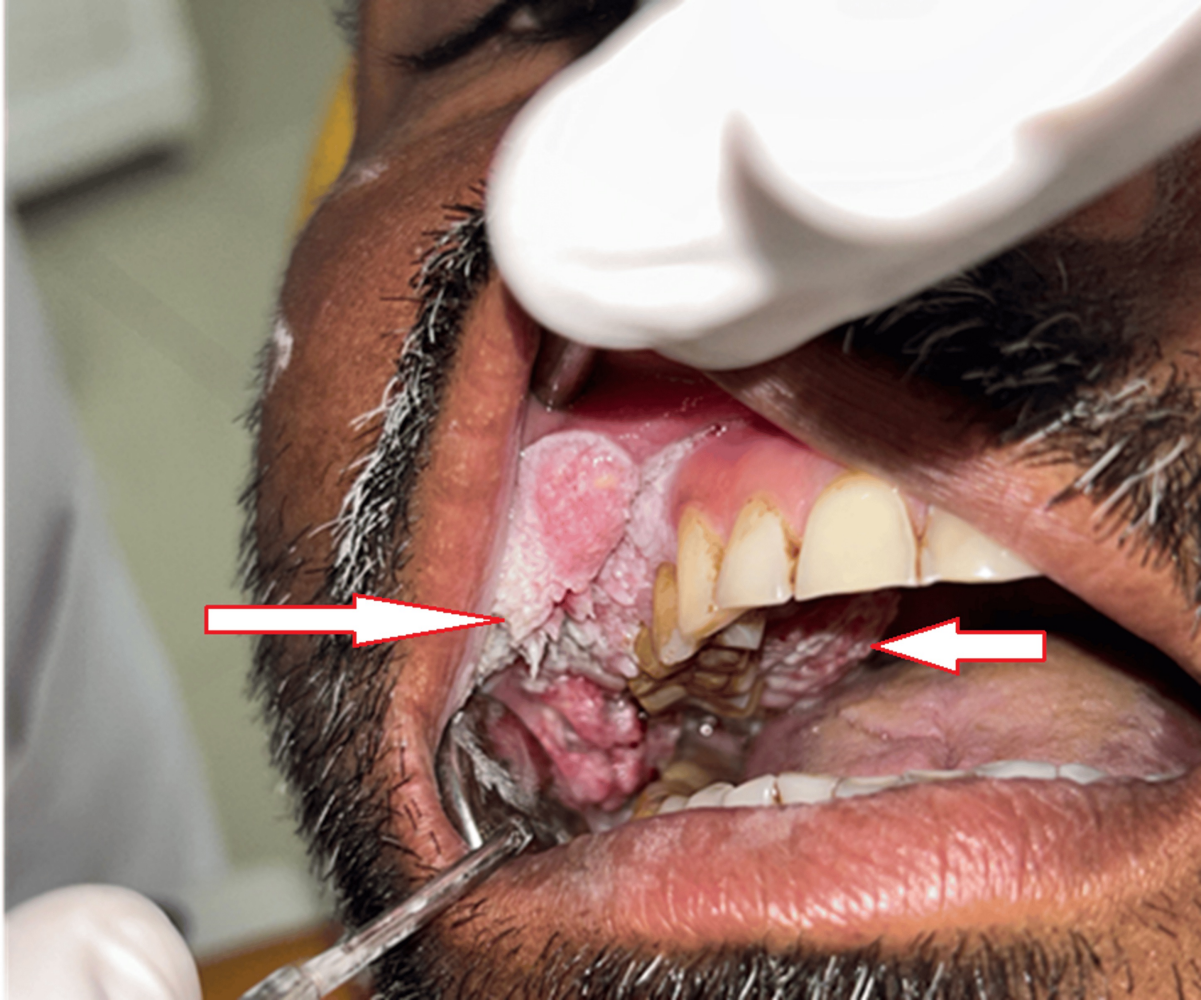
Intraoral presentation of ulcero-proliferative growth over the right maxilla and buccal mucosa

**Figure 2:**
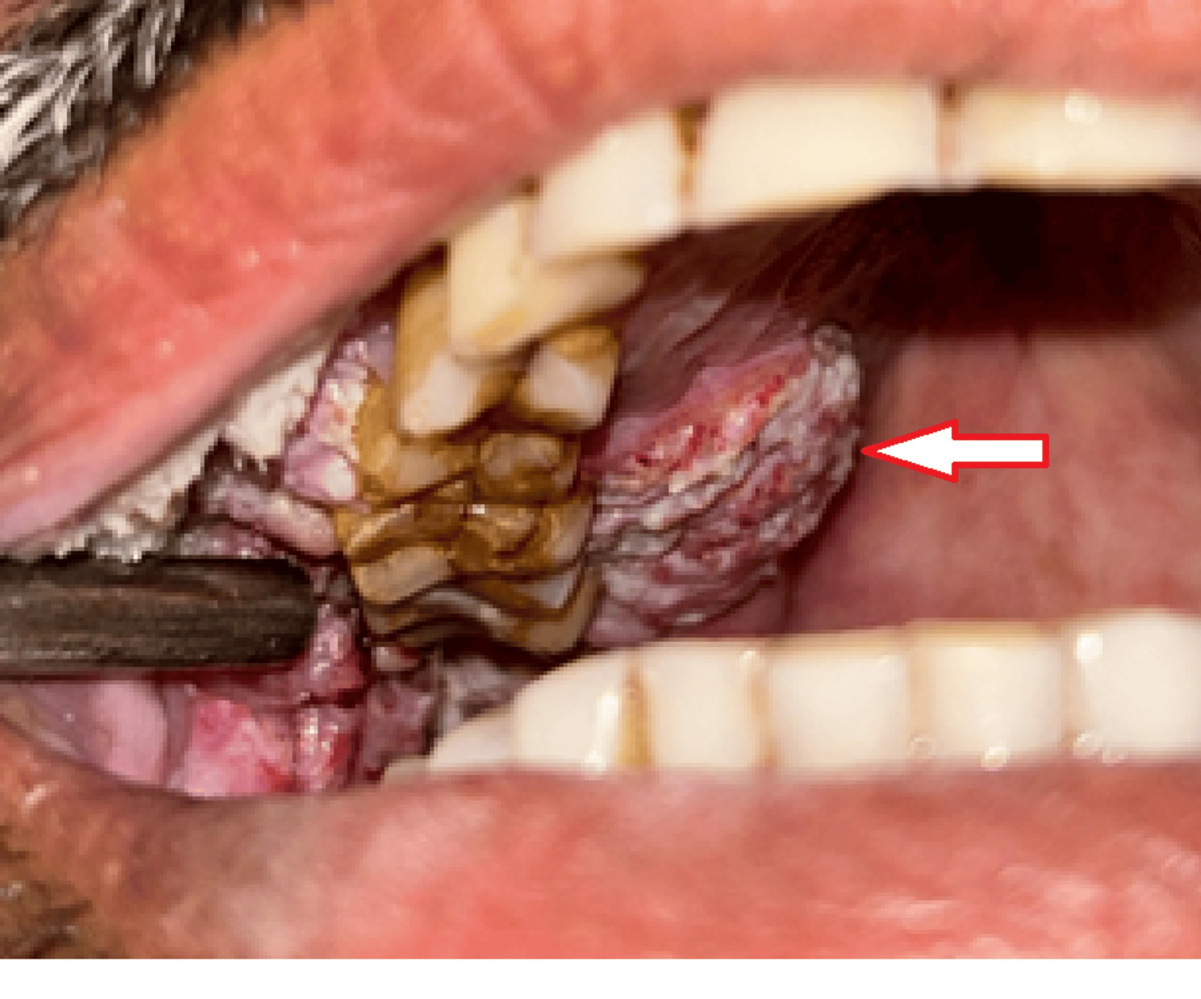
Palatal extension of the ulcero-proliferative growth

Intraorally, a well-defined keratotic mass with finger-like projections on the surface was noted over the right buccal mucosa, right maxillary alveolus, and right hard palate. Anteroposteriorly, it extended from the right corner of the mouth to the distal of the right maxillary third molar. Supero-inferiorly, it extended from 1 cm below the occlusal level of mandibular molars up to the upper right buccal vestibule. The growth also extended over the right maxillary attached gingiva on the buccal aspect of the premolars. An ulcero-proliferative growth was noted over the hard palate, extending anteroposteriorly from the right maxillary second premolar to the distal of the right maxillary third molar and mediolaterally extending from 1 cm lateral to the midline up to the crestal palatal gingiva of right maxillary molars. The lesion had two characteristic appearances: anteriorly warty, papillomatous surface and posteriorly rough, irregular surface (Figures 1, 2).

**Figure 3:**
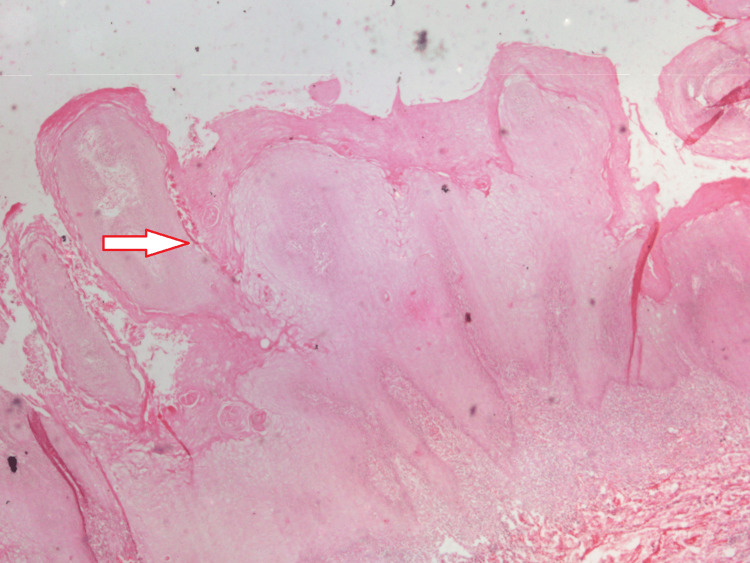
A biopsy image of the lesion. The image shown is in 10× magnification and stained with hematoxylin & eosin (H&E), demonstrating para-keratin plugging

The color is erythematous-white. The lesion measures approximately 5 × 4 cm buccally and 3 × 2 cm palatally. The surrounding area was blanched and hypopigmented. On palpation, all inspectory findings were confirmed; the lesion was non-scrapable, non-tender, had no bleeding on touch, and was non-indurated. A repeat biopsy was performed, which showed classical elongated rete pegs with pushing borders and keratin plug formations with minimal atypia of the epithelial cells, suggestive of VC (Figure 3). Oral VC lacks specific diagnostic markers [13] and is diagnosed by the method of elimination. Consequently, additional immunohistochemistry tests were not performed, as the gold standard of treatment remains surgical excision of the growth.

**Figure 4:**
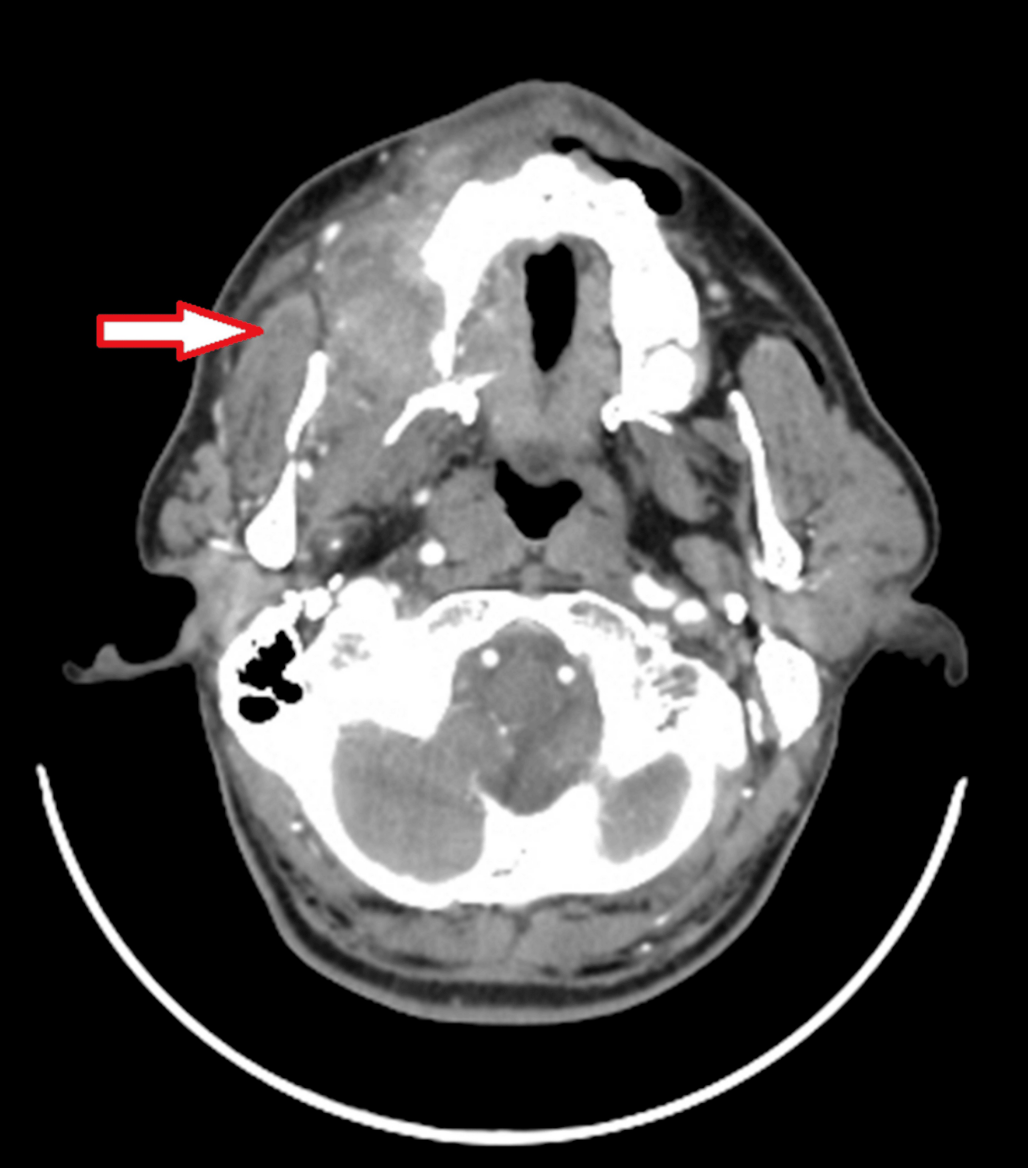
Contrast-enhanced computed tomography image of the lesion

On contrast-enhanced computed tomography (CECT), a moderately, heterogeneously enhancing ill-defined, soft tissue attenuation lesion was seen involving the right buccal mucosa with involvement of the superior gingivo-buccal sulcus and retromolar trigone along with the hard palate and medial aspect of the right maxillary alveolus and the presence of a few subcentimetric enlarged homogenous lymph nodes at levels IA, IB, II, and III, suggestive of neoplastic etiology (Figure 4).

**Figure 5:**
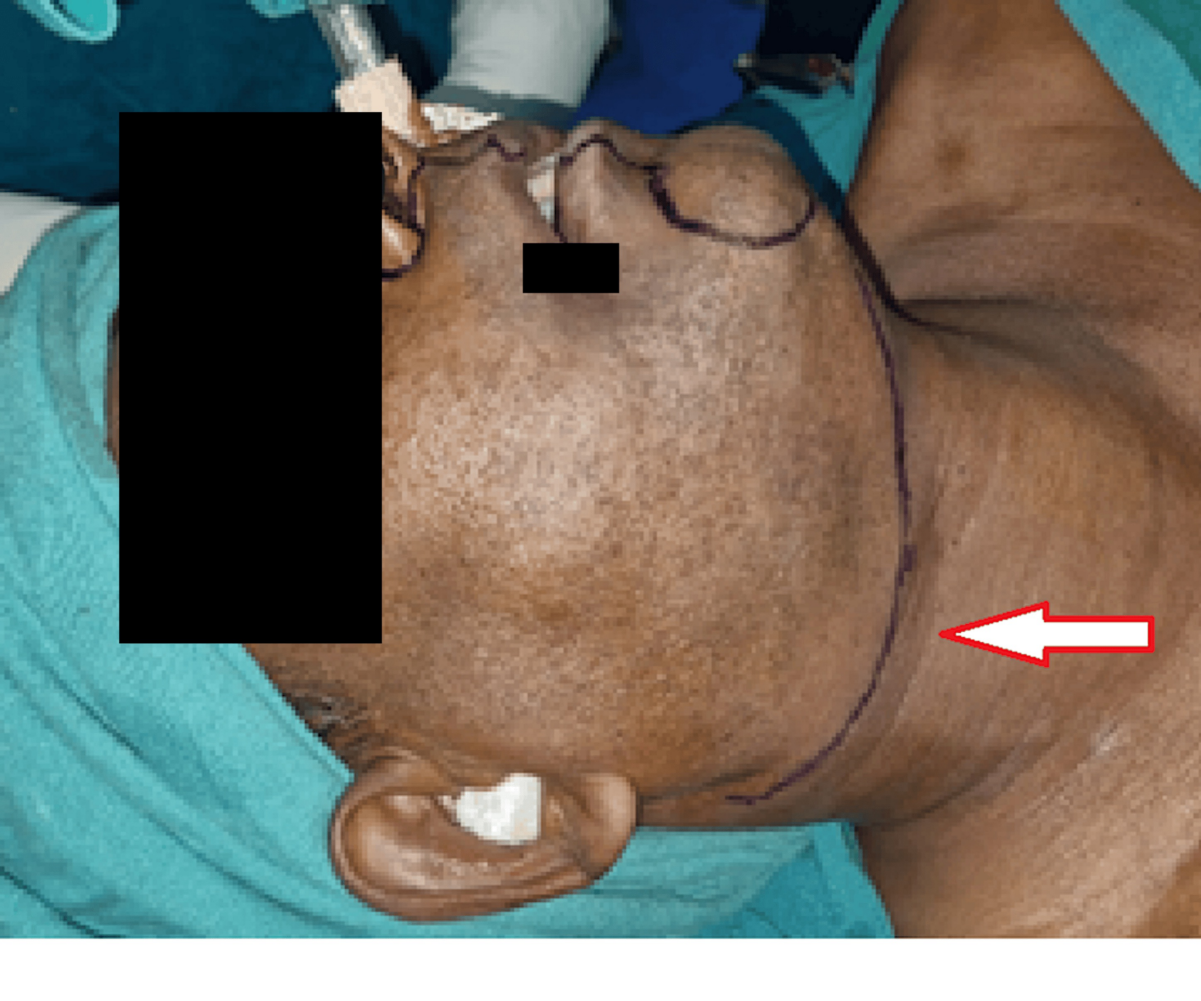
Surgical marking of the lower lip split approach

A diagnosis of VC was established based on clinical presentation, imaging, and histological findings, and the patient was scheduled for wide local excision of the growth under general anesthesia. A lower lip split approach was preferred in this case (Figure 5).

**Figure 6:**
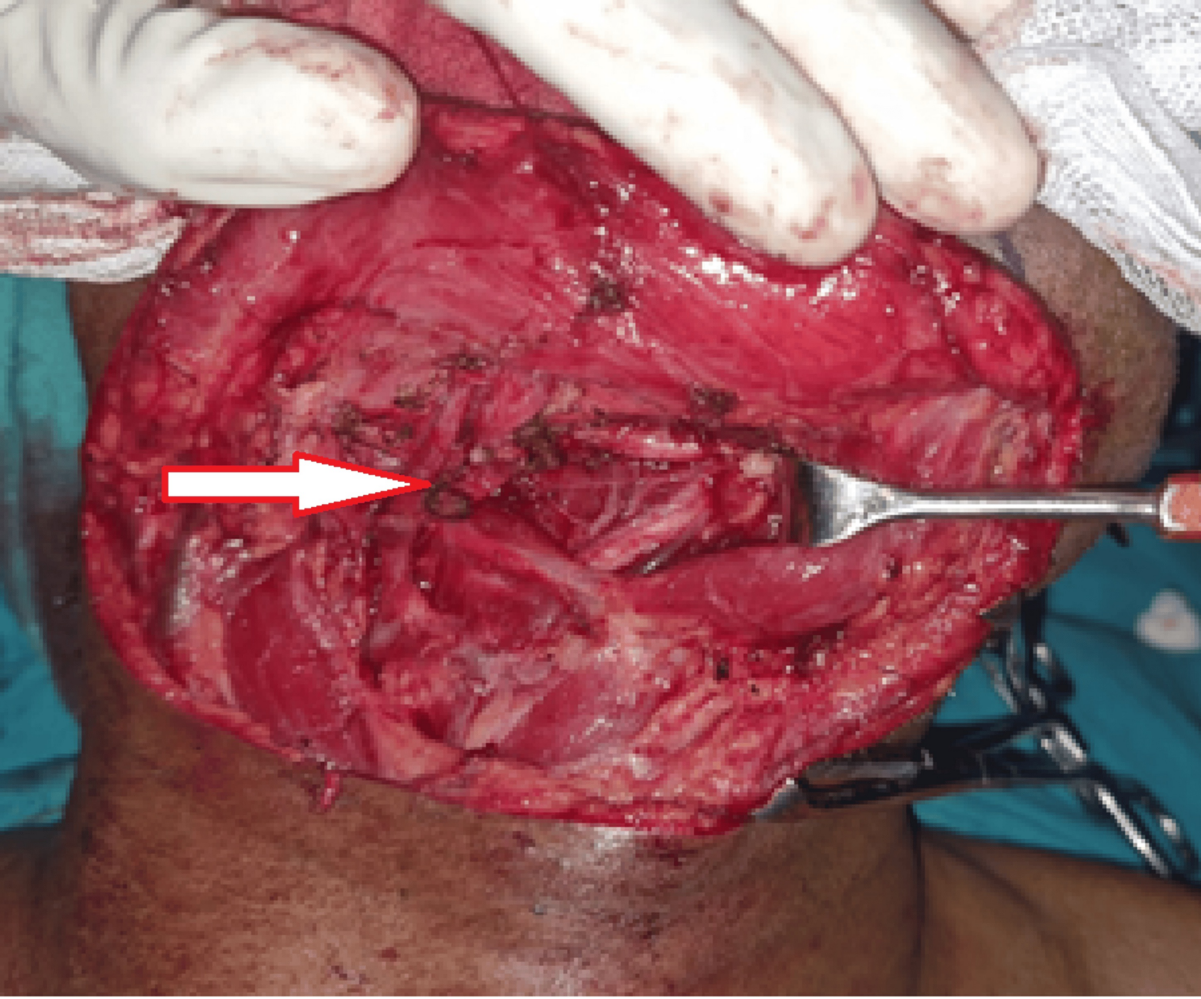
Ipsilateral supraomohyoid neck dissection and facial vessels (see arrow) preservation for microvascular anastomosis for reconstruction

The angle split approach was avoided as the lesion involved part of the right oral commissure, and leaving an adequate margin post-resection would have proved difficult. A supraomohyoid neck dissection was done on the ipsilateral side. The facial artery and vein were preserved for microvascular anastomosis for reconstruction (Figure 6).

**Figure 7:**
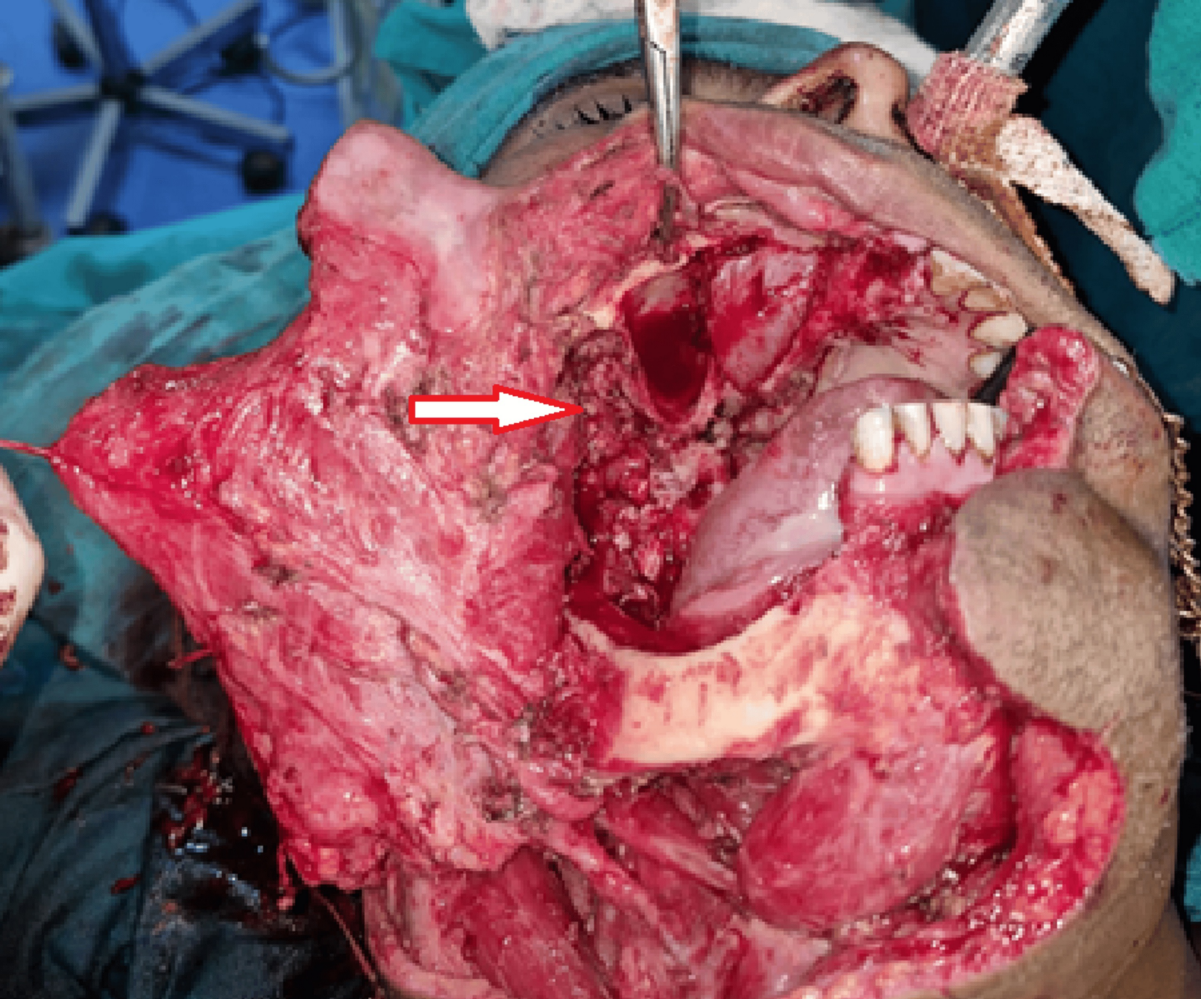
Intraoperative image of the site of the lesion after bite resection and right maxillectomy. The arrow demonstrates the defect

**Figure 8:**
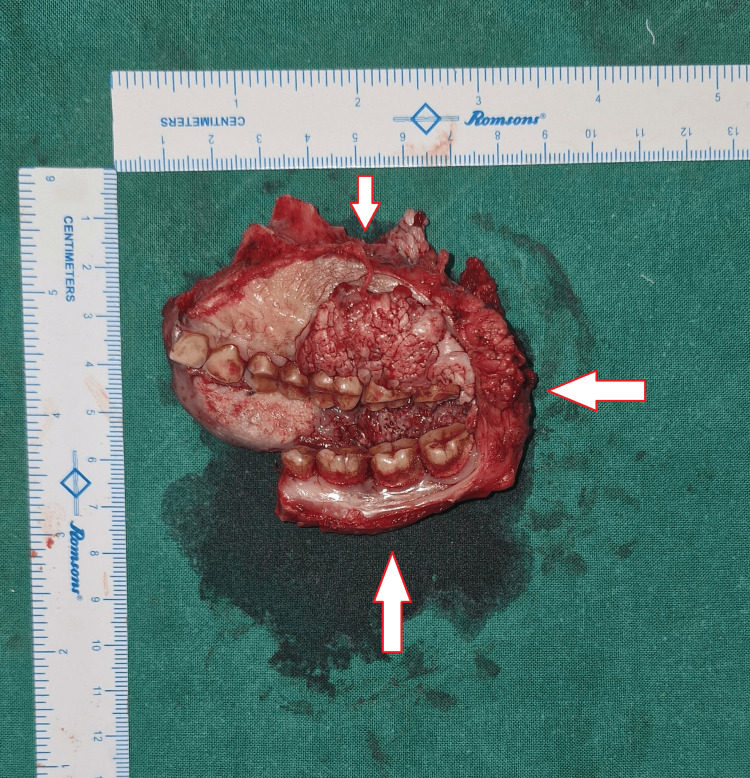
Image of the resected specimen of the lesion showing adequate safety margins

A wide local excision was done, and through the same approach, a right infrastructure maxillectomy (sparing the infraorbital rim and orbit) and a right marginal mandibulectomy (bite resection) were performed (Figures 7, 8).

**Figure 9:**
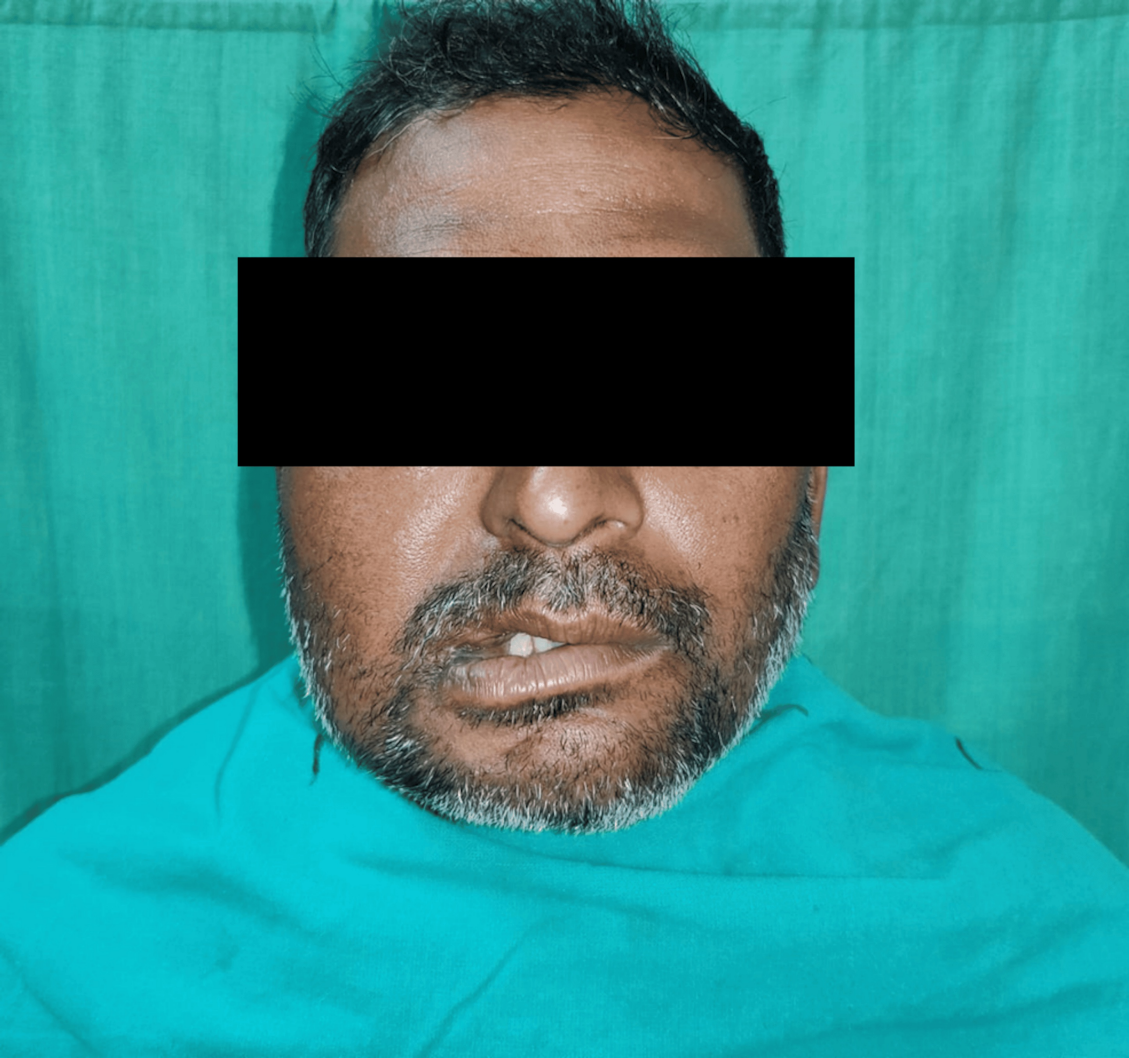
Image taken four months post-surgery

**Figure 10:**
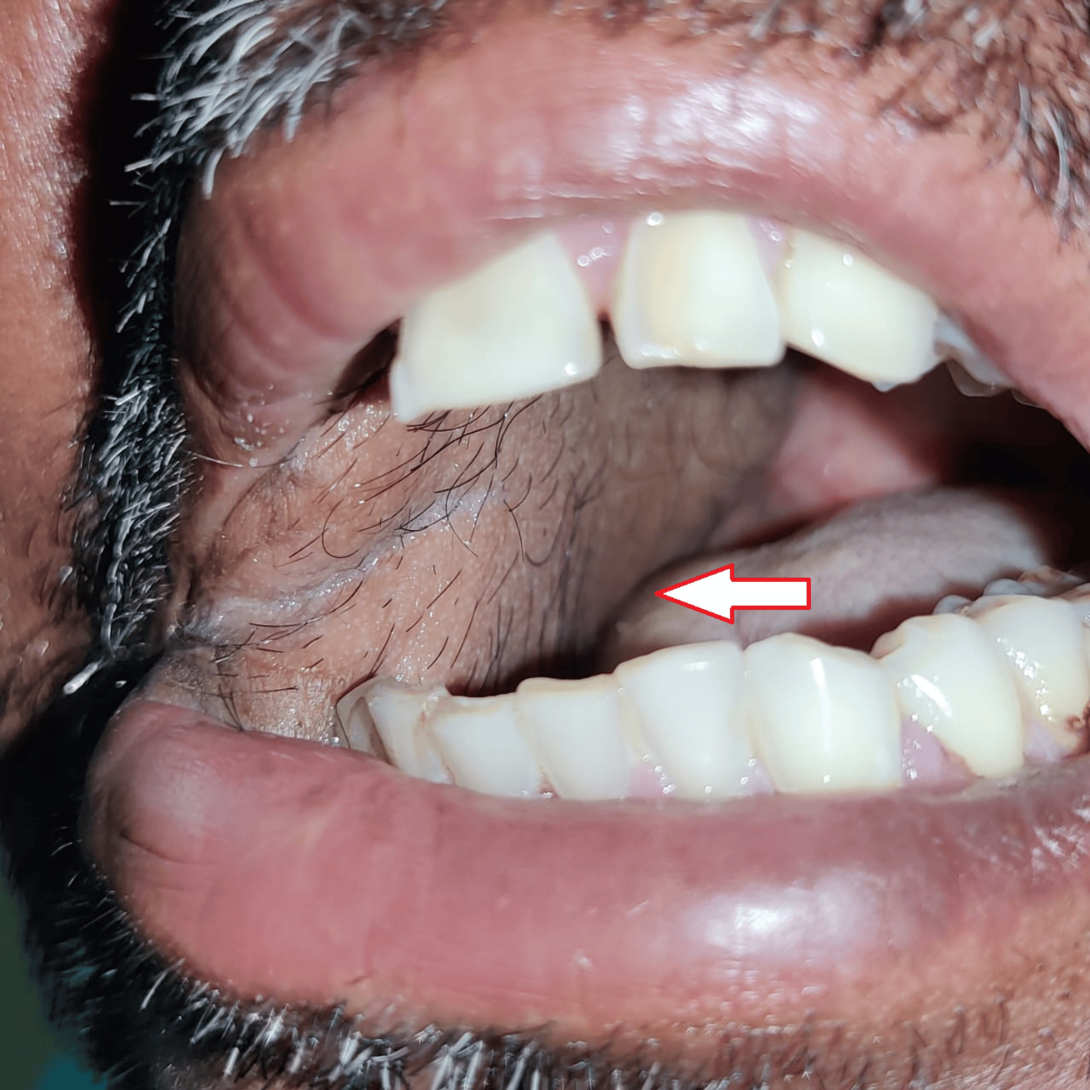
Four months postoperative image: intraoral view showing a healthy flap

Reconstruction of the defect was done using an anterolateral thigh (ALT) flap. The choice of ALT flap was made over a free fibula graft, considering a large defect, less inclination of the patient toward postoperative dental rehabilitation, and unwillingness to harvest an osseocutaneous flap. Standard postoperative protocol, such as postoperative antibiotics, analgesics, nasogastric tube feeding, drain care, and a total of eight days of postoperative hospitalization, was followed for this patient. Additionally, no adjuvant therapy, such as radiotherapy, was advised as it is not indicated for VC. The patient was kept on regular follow-up and showed satisfactory healing (Figures 9, 10).

## Discussion

VC is an uncommon, low-grade, well-differentiated form of SCC affecting the skin or mucosa. It is characterized by a verrucous or cauliflower-like appearance. This tumor exhibits locally aggressive behavior but has a low potential for metastasis, mild dysplasia, and a generally favorable prognosis. It tends to erode tissues rather than deeply infiltrate them, primarily demonstrating horizontal growth and lacking distant metastasis. Depending on the anatomical site involved, it is also known by various names, such as Ackerman tumor and carcinoma cuniculatum [15].

Sciubba and Helman outline the site predilection and frequency of occurrence of oral VC as follows: "alveolar ridge 66.6%, tongue 50%, buccal mucosa 41.6%, gingiva 33.3%, floor of the mouth 25%, labial mucosa 16.6%, and hard and soft palate 8.3% each [16].

Histologically, this condition is marked by a hyperplastic lesion exhibiting hyper-orthokeratosis and/or parakeratosis within the epithelium, along with a verrucous or papilliform surface that forms ridges. Acanthosis is commonly present, with rapid epithelial growth projecting toward the connective tissue in a test-tube-like pattern. These histopathological features were observed in our report. The basal membrane remains intact, with preserved stratification and minimal evidence of connective tissue invasion by individual tumor cells. Mitosis, pleomorphism, and hyperchromatism are rare [17].

Various surgical approaches for maxillectomy have been described in the literature, namely, the Weber-Ferguson approach, Dieffenbach's modification of the Weber-Ferguson approach, the lateral rhinotomy approach, etc. Weber-Ferguson and lateral rhinotomy approaches provide excellent surgical access; however, they require a facial incision and lip split, which is undesirable, particularly for the extirpation of benign tumors [18]. The surgical approach in this case was a lip split-modified apron flap. The lesion over the maxilla was accessed intraorally through this same incision. This unique approach avoided the use of the Weber-Ferguson incision (upper cheek flap approach) to access the maxilla for maxillectomy. Therefore, an additional scar was avoided. However, a disadvantage of this approach was that the mental nerve could not be preserved. The maxillectomy in this case, according to Brown's classification, was categorized as Class II A, indicating a vertical component: low maxillectomy, which involves the resection of the maxillary alveolus and the antral walls and not the orbital rim and floor, and a horizontal component, which is the unilateral resection of the alveolar maxilla and hard palate.

## Conclusions

VC, a rare and distinct subtype of well-differentiated SCC, poses unique diagnostic and therapeutic challenges due to its indolent yet locally aggressive behavior and its potential to mimic other pathological lesions. The surgical management involving wide local excision, ipsilateral supra-omohyoid neck dissection, and partial maxillectomy with a marginal mandibulectomy underscores the importance of obtaining clear margins to prevent recurrence. The use of an innovative lip split apron flap for surgical access ensured optimal exposure while minimizing additional scarring, demonstrating the value of customized approaches in complex cases.

This case highlights the necessity of an interdisciplinary approach, combining precise surgical planning, robust diagnostic tools, and reconstructive expertise, to achieve favorable outcomes in managing VC.
